# Supplementary material for: Spin-dependent phenomena at chiral temporal interfaces
Source: Nanophotonics. 2023 Mar 16;12(14):2881–9. doi: 10.1515/nanoph-2022-0805 (PMC11501299; doi:10.1515/nanoph-2022-0805)
Supplement: Supplementary file 1 — Supplementary Material Details [file j_nanoph-2022-0805_suppl.pdf]

Supplementary Materials
Spin-dependent phenomena at chiral temporal interfaces

M. H. Mostafa*, M. S. Mirmoosa, S. A. Tretyakov*
Department of Electronics and Nanoengineering, Aalto University, Espoo, Finland
* Correspondence: mohamed.mostafa@aalto.fi, sergei.tretyakov@aalto.fi

Materials and methods

The time-domain solver of the commercial software COMSOL Multiphysics® has been used to numerically simulate temporal interfaces. The simulation setup (Fig. 3A) has dimensions equal to $14\lambda_\mathrm{d} \times 0.7\lambda_\mathrm{d}$, where λ_d is the wavelength in the dielectric medium. A triangular mesh is implemented with minimum and maximum dimensions of 3×10^{-5} m and $\lambda_\mathrm{d}/20$. Periodic boundary conditions are applied at the top and bottom boundaries, to emulate plane waves. The boundary on the left is assigned to a scattering boundary behaving as a source, and the right boundary is assigned to a scattering boundary behaving as a perfect absorber. Electric field of circularly polarized plane waves propagating in the x-direction is excited using an analytical expression $\mathbf{E}^{\pm} = \frac{E_0}{2}\left[\hat{\mathbf{y}} \mp j\hat{\mathbf{z}}\right] e^{j(\omega t - \beta^{\pm} x)}$. The temporal interfaces are modeled by inducing fast but smooth changes in the permittivity and permeability filling the rectangular box. Initially, the medium has permitivity $\epsilon_\mathrm{eq}^{\pm}$ and permeability $\mu_\mathrm{eq}^{\pm}$, then they change following a rectangular shape using analytical functions with smooth transitions with two continuous derivatives, while the duration of the transient period is 1.1^{-10} s.

Supplementary Note 1

Time-domain material relations in chiral media

To study time-varying chiral media, time-domain constitutive relations are needed. The commonly used constitutive equations of chiral media (so called Post and Tellegen relations) (*27*) are applicable only in the frequency domain. Because the chirality parameter in these formalisms is inherently frequency dispersive, transforming these equations to time domain would complicate the mathematical formulation, as the material relations would contain convolution integrals. Thus, Condon model is used (*28*), which approximately models non-resonant chirality effects with a non-dispersive parameter g. In chiral media, a linearly polarized plane wave can be expressed as a combination of RHCP and LHCP waves having the same angular frequency but propagating at different phase velocities. Splitting the fields of a plane wave into RHCP and LHCP components, and following the Condon model, we write the constitutive relations of isotropic chiral media as

$$\mathbf{D} = \overbrace{\left[\epsilon_{\text{eff}}\mathbf{E}^{-} + g\frac{\partial \mathbf{H}^{-}}{\partial t}\right]}^{\mathbf{D}^{-}} + \overbrace{\left[\epsilon_{\text{eff}}\mathbf{E}^{+} + g\frac{\partial \mathbf{H}^{+}}{\partial t}\right]}^{\mathbf{D}^{+}}, \tag{S1a}$$

$$\mathbf{B} = \overbrace{\left[\mu_{\text{eff}}\mathbf{H}^{-} - g\frac{\partial \mathbf{E}^{-}}{\partial t}\right]}^{\mathbf{B}^{-}} + \overbrace{\left[\mu_{\text{eff}}\mathbf{H}^{+} - g\frac{\partial \mathbf{E}^{+}}{\partial t}\right]}^{\mathbf{B}^{+}}, \tag{S1b}$$

in which ϵ_{eff}, μ_{eff}, and g are the non-dispersive effective permittivity, effective permeability, and the chirality parameter (or rotatory parameter as Condon called it). Electric and magnetic fields of a linearly polarized plane wave propagating in x-direction is considered, and the fields read

$$\mathbf{E}^{\pm} = \frac{E_0}{2}\left[\cos(\omega t - \beta^{\pm}x)\hat{\mathbf{y}} \pm \sin(\omega t - \beta^{\pm}x)\hat{\mathbf{z}}\right], \tag{S2a}$$

$$\mathbf{H}^{\pm} = \frac{E_0}{2\eta_{\text{eff}}}\left[\mp\sin(\omega t - \beta^{\pm}x)\hat{\mathbf{y}} + \cos(\omega t - \beta^{\pm}x)\hat{\mathbf{z}}\right], \tag{S2b}$$

where η_{eff} is the medium effective intrinsic impedance, and E_0 is the amplitude of the electric field. Considering the fields derivatives $\frac{\partial \mathbf{H}^{\pm}}{\partial t} = \frac{\mp\omega}{\eta_{\text{eff}}}\mathbf{E}^{\pm}$ and $\frac{\partial \mathbf{E}^{\pm}}{\partial t} = \pm\omega\eta_{\text{eff}}\mathbf{H}^{\pm}$, Eqs. (S1a) and

(S1b) can be expressed as

$$\mathbf{D} = \Bigg[\overbrace{\epsilon_{\text{eff}}\mathbf{E}^- + \frac{g\omega}{\eta_{\text{eff}}}\mathbf{E}^-}^{\mathbf{D}^-}\Bigg] + \Bigg[\overbrace{\epsilon_{\text{eff}}\mathbf{E}^+ - \frac{g\omega}{\eta_{\text{eff}}}\mathbf{E}^+}^{\mathbf{D}^+}\Bigg], \tag{S3a}$$

$$\mathbf{B} = \Bigg[\overbrace{\mu_{\text{eff}}\mathbf{H}^- + g\omega\eta_{\text{eff}}\mathbf{H}^-}^{\mathbf{B}^-}\Bigg] + \Bigg[\overbrace{\mu_{\text{eff}}\mathbf{H}^+ - g\omega\eta_{\text{eff}}\mathbf{H}^+}^{\mathbf{B}^+}\Bigg], \tag{S3b}$$

which simplifies to

$$\mathbf{D}^\pm = \epsilon_{\text{eff}}\Big(1 \mp \Psi\Big)\mathbf{E}^\pm, \tag{S4a}$$

$$\mathbf{B}^\pm = \mu_{\text{eff}}\Big(1 \mp \Psi\Big)\mathbf{H}^\pm, \tag{S4b}$$

where $\Psi = g\omega c$, ω is the angular frequency, and $c = \frac{1}{\sqrt{\epsilon_{\text{eff}}\mu_{\text{eff}}}}$. We note that the above relations are in a similar form as the relations for dispersive stationary chiral media, however, the models used to arrive to both relations are different and cannot replace each other.

Supplementary Note 2

Temporal boundary conditions

A. Chiral-dielectric temporal interface

We contemplate a chiral medium supporting a linearly polarized plane wave at frequency ω_1 when the non-dispersive chirality parameter g rapidly changes to zero, that is, the medium becomes nonchiral. In his paper published in 1958, Morgenthaler showed that the electric and magnetic flux densities are continuous at a temporal interface (*29*). Using that property, we write that $\mathbf{D}_1^{\pm} = \mathbf{D}_2^{\pm}$ and $\mathbf{B}_1^{\pm} = \mathbf{B}_2^{\pm}$, where the subscripts $1, 2$ correspond to the fields before $(t = t_0^-)$ and after $(t = t_0^+)$ the temporal discontinuity, respectively (t_0 is the switching moment). The flux densities at t_0^- equal $\mathbf{D}_1^{\pm} = \epsilon_{\text{eff}}\Big(1 \mp \Psi_1\Big)\mathbf{E}_1^{\pm}$ and $\mathbf{B}_1^{\pm} = \mu_{\text{eff}}\Big(1 \mp \Psi_1\Big)\mathbf{H}_1^{\pm}$, in which $\Psi_1 = \omega_1 g c$. While at t_0^+ they equal $\mathbf{D}_2^{\pm} = \epsilon_{\text{eff}}\mathbf{E}_2^{\pm}$ and $\mathbf{B}_2^{\pm} = \mu_{\text{eff}}\mathbf{H}_2^{\pm}$. According to Morgenthaler, after the temporal jump, there are forward and backward waves in analogy with a spatial interface at which we have transmitted and reflected waves. As a result, the fields after the temporal interface can be written as

$$
\begin{aligned}
\mathbf{E}_2^{\pm} &= \Upsilon_{\text{c}\|\text{d}}^{\pm}\frac{E_0}{2}\Bigg[\cos(\omega_2^{\pm}t - \beta_2^{\pm}x)\hat{\mathbf{y}} \pm \sin(\omega_2^{\pm}t - \beta_2^{\pm}x)\hat{\mathbf{z}}\Bigg] \\
&+ \Gamma_{c\|d}^{\pm}\frac{E_0}{2}\Bigg[\cos(\omega_2^{\pm}t + \beta_2^{\pm}x)\hat{\mathbf{y}} \mp \sin(\omega_2^{\pm}t + \beta_2^{\pm}x)\hat{\mathbf{z}}\Bigg], \qquad \text{(S5a)} \\
\mathbf{H}_2^{\pm} &= \frac{\Upsilon_{\text{c}\|\text{d}}^{\pm}E_0}{2\eta_{\text{eff}}}\Bigg[\cos(\omega_2^{\pm}t - \beta^{\pm}x)\hat{\mathbf{z}} \mp \sin(\omega_2^{\pm}t - \beta^{\pm}x)\hat{\mathbf{y}}\Bigg] \\
&- \frac{\Gamma_{c\|d}^{\pm}E_0}{2\eta_{\text{eff}}}\Bigg[\cos(\omega_2^{\pm}t + \beta^{\pm}x)\hat{\mathbf{z}} \pm \sin(\omega_2^{\pm}t + \beta^{\pm}x)\hat{\mathbf{y}}\Bigg]. \qquad \text{(S5b)}
\end{aligned}
$$

The forward and backward propagation coefficients (for a temporal interface between chiral and dielectric media) are denoted as $\Upsilon_{\text{c}\|\text{d}}$ and $\Gamma_{\text{c}\|\text{d}}$, respectively. The backward propagating wave is defined by the negative frequency component after the temporal interface. Interestingly,

both the forward and backward waves keep the same polarization state, resulting in handedness conservation along the temporal interface. Assuming that $t_0 = 0$, the fields reduce to

$$\mathbf{E}_1^{\pm} = \frac{E_0}{2}\left[\cos(\beta_1^{\pm}x)\hat{\mathbf{y}} \mp \sin(\beta_1^{\pm}x)\hat{\mathbf{z}}\right], \tag{S6a}$$

$$\mathbf{E}_2^{\pm} = (\Upsilon_{\mathrm{c\|d}}^{\pm} + \Gamma_{c\|d}^{\pm})\frac{E_0}{2}\left[\cos(\beta_2^{\pm}x)\hat{\mathbf{y}} \mp \sin(\beta_2^{\pm}x)\hat{\mathbf{z}}\right], \tag{S6b}$$

$$\mathbf{H}_1^{\pm} = \frac{E_0}{2\eta_{\mathrm{eff}}}\left[\cos(\beta^{\pm}x)\hat{\mathbf{z}} \pm \sin(\beta^{\pm}x)\hat{\mathbf{y}}\right], \tag{S6c}$$

$$\mathbf{H}_2^{\pm} = (\Upsilon_{\mathrm{c\|d}}^{\pm} - \Gamma_{c\|d}^{\pm})\frac{E_0}{2\eta_{\mathrm{eff}}}\left[\cos(\beta^{\pm}x)\hat{\mathbf{z}} \pm \sin(\beta^{\pm}x)\hat{\mathbf{y}}\right]. \tag{S6d}$$

The spatial frequency is conserved at the temporal interface as no spatial boundaries are introduced, leading to $\beta_1^{\pm} = \beta_2^{\pm}$. Then we find that $\frac{\mathbf{E}_2^{\pm}}{\mathbf{E}_1^{\pm}} = (\Upsilon_{\mathrm{c\|d}}^{\pm} + \Gamma_{c\|d}^{\pm})$ and $\frac{\mathbf{H}_2^{\pm}}{\mathbf{H}_1^{\pm}} = (\Upsilon_{\mathrm{c\|d}}^{\pm} - \Gamma_{c\|d}^{\pm})$. From $\mathbf{D}_1^{\pm} = \mathbf{D}_2^{\pm}$ and $\mathbf{B}_1^{\pm} = \mathbf{B}_2^{\pm}$ we get $\frac{\mathbf{E}_2^{\pm}}{\mathbf{E}_1^{\pm}} = 1 \mp \Psi_1$ and $\frac{\mathbf{H}_2^{\pm}}{\mathbf{H}_1^{\pm}} = 1 \mp \Psi_1$. Finally, we see that $\Upsilon_{\mathrm{c\|d}}^{\pm} + \Gamma_{c\|d}^{\pm} = 1 \mp \Psi_1$ and $\Upsilon_{\mathrm{c\|d}}^{\pm} - \Gamma_{c\|d}^{\pm} = 1 \mp \Psi_1$, leading to $\Gamma_{c\|d}^{\pm} = 0$.

B. Chiral temporal slab

To form a chiral temporal slab, another temporal interface between dielectric and chiral media has to take place. The material parameters after the second temporal interface are $\epsilon_{\mathrm{eq}} = \epsilon_{\mathrm{eff}}(1 \mp \Psi_3)$, $\mu_{\mathrm{eq}} = \mu_{\mathrm{eff}}(1 \mp \Psi_3)$, and $g \neq 0$, where $\Psi_3 = g\omega_3^{\pm}c$. The forward and backward propagation coefficients can be calculated by the Morgenthaler equations, leading to $\Upsilon_{d\|c}^{\pm} = \frac{1}{1 \mp \Psi_3^{\pm}}$ and $\Gamma_{d\|c}^{\pm} = 0$. The angular frequencies $\omega_3^{\pm}$ can be calculated from the conservation of phase constants $\beta_1^{\pm} = \beta_2^{\pm} = \beta_3^{\pm}$, where $\beta_3^{\pm} = \omega_3^{\pm}\sqrt{\mu_{\mathrm{eff}}\epsilon_{\mathrm{eff}}}(1 \mp \Psi_3^{\pm})$. By solving for $\omega_3^{\pm}$ from $\beta_2^{\pm} = \beta_3^{\pm}$ we get

$$\omega_3^{\pm} = \frac{\pm 1 \pm \sqrt{1 \mp 4g\omega_2^{\pm}c}}{2cg}. \tag{S7}$$

Substituting $\omega_2^{\pm} = \omega_1(1 \mp \Psi_1)$ leads to

$$\omega_3^+ = \frac{\frac{1}{2} \pm |(\Psi_1 - \frac{1}{2}|}{cg}, \tag{S8a}$$

$$\omega_3^- = \frac{\frac{1}{2} \pm |\Psi_1 + \frac{1}{2}|}{cg}, \tag{S8b}$$

in which $\pm$ represent the positive and negative branches of the square root. Considering $\Psi_1 < \frac{1}{2}$, we get

$$\omega_3^+ = \begin{cases} \frac{\frac{1}{2}+\Psi_1-\frac{1}{2}}{cg}, & \text{for negative solution} \\ \frac{\frac{1}{2}-\Psi_1+\frac{1}{2}}{cg}, & \text{for positive solution} \end{cases} \tag{S9a}$$

$$\omega_3^- = \begin{cases} \frac{-\frac{1}{2}-\Psi_1-\frac{1}{2}}{cg}, & \text{for negative solution} \\ \frac{-\frac{1}{2}+\Psi_1+\frac{1}{2}}{cg}, & \text{for positive solution} \end{cases} \tag{S9b}$$

which simplifies to

$$\omega_3^+ = \begin{cases} \omega_1^+, & \text{for negative solution} \\ \frac{1}{cg} - \omega_1^+, & \text{for positive solution} \end{cases} \tag{S10a}$$

$$\omega_3^- = \begin{cases} -\frac{1}{cg} - \omega_1^-, & \text{for negative solution} \\ \omega_1^-, & \text{for positive solution} \end{cases} \tag{S10b}$$

The chiral composite considered in the main text had chirality parameter $g = 1.0348 \times 10^{-20}\ \mathrm{s}^2/\mathrm{m}$ and $\omega_1/(2\pi) = 3$ GHz. Hence, the solution $| \pm \frac{1}{cg} - \omega_1^{\pm}|$ results in $\omega_3^{\pm}$ being much larger than the resonance frequency. At such frequencies the time-domain Condon model is not applicable. Thus, only solution $\omega_3^{\pm} = \omega_1^{\pm}$ is considered and discussed in the main text.

Supplementary Note 3

Effective parameters of uniaxial chiral composite

Here we consider microwave uniaxial chiral media composits in details. Consider a canonical metal-wire left-handed chiral particle, where the particle is formed by two short straight wires (the arm length l) connected to an electrically small loop (the loop radius a) (Fig. S1). For electrically small lossless wire antennas and loops the input impedances can be approximated as $Z_{\mathrm{wire}} = \frac{1}{j\omega C}$ and $Z_{\mathrm{loop}} = j\omega L$, where C and L are the capacitance of the wire and the inductance of the loop that are approximately equal to (*30*)

$$C = \frac{\pi l \epsilon}{\ln(\frac{2l}{r_0})}, \tag{S11a}$$

$$L = \mu a \left[\ln\left(\frac{8a}{r_0}\right) - 2 \right], \tag{S11b}$$

where ϵ and μ are the permitivity and permeability of the background medium, and r_0 is the radius of the wire. The polarizability dyadics for this particle are expressed as

$$\overline{\overline{\alpha}}_{\mathrm{ee}} = \alpha_{\mathrm{ee}}^{\mathrm{zz}}\hat{\mathbf{z}}\hat{\mathbf{z}} + \alpha_{\mathrm{ee}}^{\mathrm{xx}}\hat{\mathbf{x}}\hat{\mathbf{x}} + \alpha_{\mathrm{ee}}^{\mathrm{yy}}\hat{\mathbf{y}}\hat{\mathbf{y}} + \alpha_{\mathrm{ee}}^{\mathrm{yz}}\hat{\mathbf{y}}\hat{\mathbf{z}} + \alpha_{\mathrm{ee}}^{\mathrm{zy}}\hat{\mathbf{z}}\hat{\mathbf{y}}, \tag{S12a}$$

$$\overline{\overline{\alpha}}_{\mathrm{mm}} = \alpha_{\mathrm{mm}}^{\mathrm{zz}}\hat{\mathbf{z}}\hat{\mathbf{z}}, \tag{S12b}$$

$$\overline{\overline{\alpha}}_{\mathrm{me}} = \alpha_{\mathrm{me}}^{\mathrm{zz}}\hat{\mathbf{z}}\hat{\mathbf{z}}. \tag{S12c}$$

By choosing $2a \ll 2l$, $\alpha_{\mathrm{ee}}^{\mathrm{xx}}$ and $\alpha_{\mathrm{ee}}^{\mathrm{yy}}$ become negligible, and the cross-coupling components average to zero (*30*) in uniaxial composites, thus, the particle can be electrically polarized in the $\hat{\mathbf{z}}$ direction only. The axial polarizabilities at frequencies well below the resonance equal to

$$\alpha_{\mathrm{ee}}^{\mathrm{zz}} = \frac{l^2}{j\omega(Z_{\mathrm{wire}} + Z_{\mathrm{loop}})}, \tag{S13a}$$

$$\alpha_{\mathrm{mm}}^{\mathrm{zz}} = \frac{-j\omega\mu^2 S^2}{(Z_{\mathrm{wire}} + Z_{\mathrm{loop}})}, \tag{S13b}$$

$$\alpha_{\mathrm{me}}^{\mathrm{zz}} = \frac{-\mu S l}{(Z_{\mathrm{wire}} + Z_{\mathrm{loop}})}, \tag{S13c}$$

which are simplified to

$$\alpha_{\rm ee}^{\rm zz} = \frac{l^2 C}{1-\omega^2 LC}, \tag{S14a}$$

$$\alpha_{\rm mm}^{\rm zz} = \frac{\mu^2\omega^2 S^2 C}{1-\omega^2 LC}, \tag{S14b}$$

$$\alpha_{\rm em}^{\rm zz} = -\alpha_{\rm me}^{\rm zz} = \frac{j\omega\mu SlC}{1-\omega^2 LC}, \tag{S14c}$$

where $S=\pi a^2$ is the loop area. Next we estimate the effective material parameters of the uniaxial chiral composite using the Maxwell-Garnett model for mixtures of bianisotropic particles (*30,31*). By solving for the transversal components we get

$$\epsilon_{\rm eff} = \epsilon + \frac{1}{D_{\rm tr}}\left[N\alpha_{\rm ee} - \frac{N^2}{3\mu}(\alpha_{\rm ee}\alpha_{\rm mm}+\alpha_{\rm me}^2)\right], \tag{S15a}$$

$$\mu_{\rm eff} = \mu + \frac{1}{D_{\rm tr}}\left[N\alpha_{\rm mm} - \frac{N^2}{3\epsilon}(\alpha_{\rm ee}\alpha_{\rm mm}+\alpha_{\rm me}^2)\right], \tag{S15b}$$

$$\alpha_{\rm eff} = -\frac{N\alpha_{\rm me}}{D_{\rm tr}}, \tag{S15c}$$

where N is the number of particles per unit volume and

$$\alpha_{\rm ee} = \frac{\alpha_{\rm ee}^{\rm zz}}{2}, \tag{S16a}$$

$$\alpha_{\rm mm} = \frac{\alpha_{\rm mm}^{\rm zz}}{2}, \tag{S16b}$$

$$\alpha_{\rm me} = \frac{\alpha_{\rm me}^{\rm zz}}{2}, \tag{S16c}$$

$$D_{\rm tr} = \left(1-\frac{N\alpha_{\rm ee}}{3\epsilon}\right)\left(1-\frac{N\alpha_{\rm mm}}{3\mu}\right) + \frac{N^2\alpha_{\rm me}^2}{9\epsilon\mu}. \tag{S16d}$$

The uniaxial polarizabilities are divided by 2 as only half of the particles are polarized in one direction. Enough small N is selected, so that the expressions $\frac{N^2}{3\mu}(\alpha_{\rm ee}\alpha_{\rm mm}+\alpha_{\rm me}^2)$, $\frac{N^2}{3\epsilon}(\alpha_{\rm ee}\alpha_{\rm mm}+\alpha_{\rm me}^2)$, and $\frac{N^2\alpha_{\rm me}^2}{9\epsilon\mu}$ become negligible. As a result, switching chirality does not affect the effective permittivity and permeability. In addition, well below the particle resonance the magnetic polarizability is negligible being a second-order spatial dispersion effect, and a non-magnetic

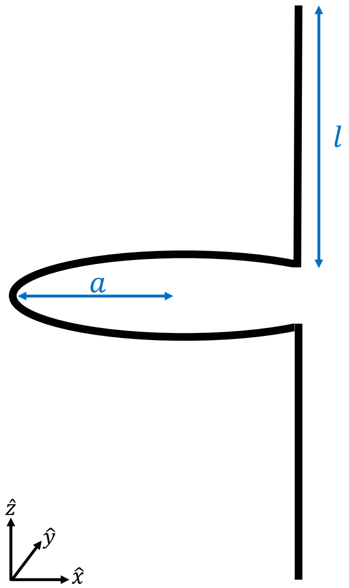

Fig. S1: Canonical metal-wire left-handed chiral particle formed by two short straight wires (arm length l) connected to an electrically small loop (the loop radius a).

background medium is considered, thus, we have $\mu_{\text{eff}} = \mu_0$. Considering these approximations, the effective parameters reduce to

$$\epsilon_{\text{eff}} = \epsilon + \frac{N\alpha_{\text{ee}}}{1 - \frac{N\alpha_{\text{ee}}}{3\epsilon}}, \tag{S17a}$$

$$\mu_{\text{eff}} = \mu_0, \tag{S17b}$$

$$\alpha_{\text{eff}} = -\frac{N\alpha_{\text{me}}}{1 - \frac{N\alpha_{\text{ee}}}{3\epsilon}}. \tag{S17c}$$

Supplementary Note 4

Extended numerical results

Figure S2 shows the z-component of electric field and the y- and z-components of the magnetic field. All field components get affected by the spin-dependent phenomenon, as expected.

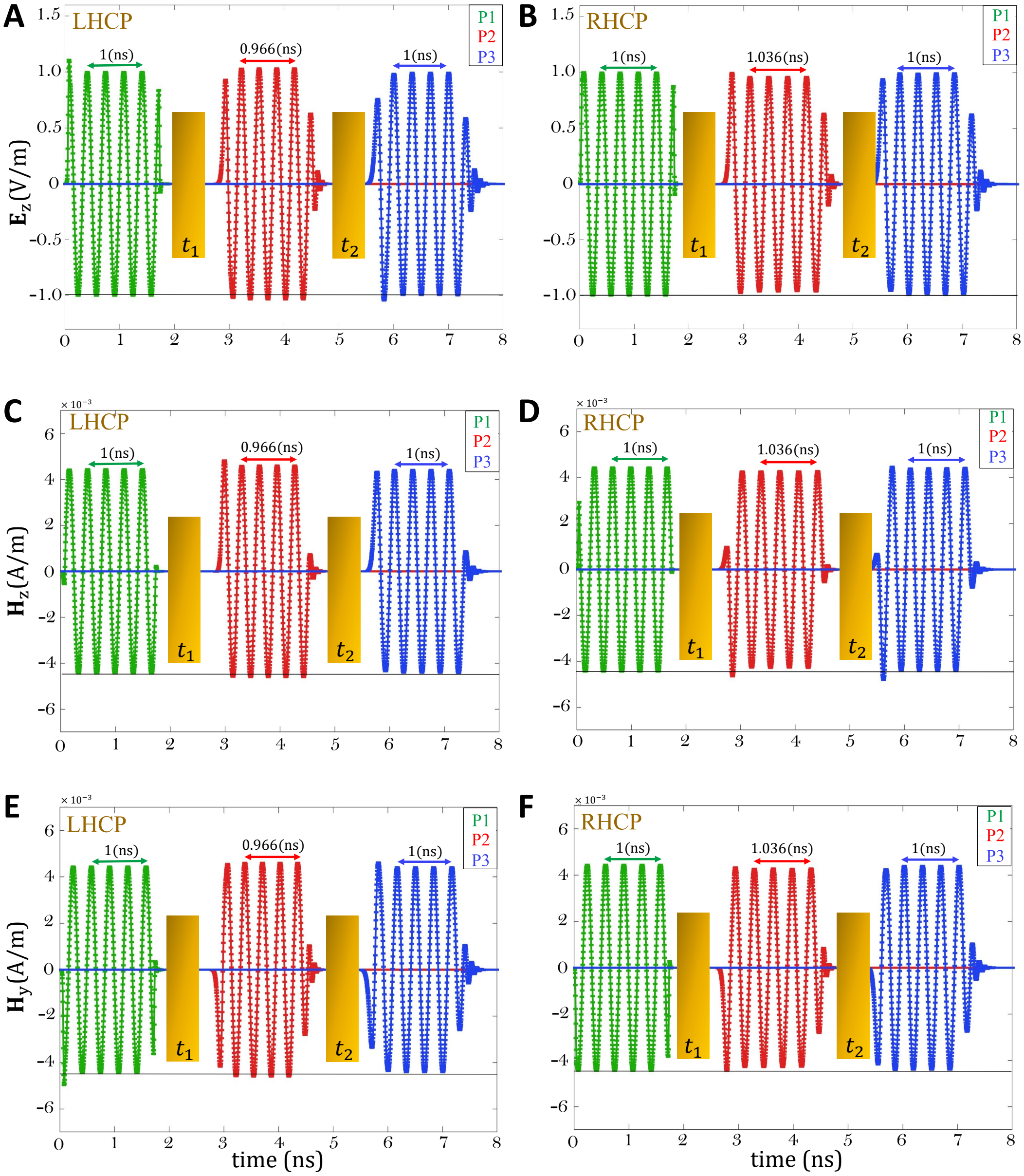

Fig. S2: Extended numerical results for the numerical simulations presented in the main text. (A)-(B) z-component of electric field. (C)-(F) y- and z-components of the magnetic field.
